# Graphene oxide–iridium nanocatalyst for the transformation of benzylic alcohols into carbonyl compounds

**DOI:** 10.1039/c9ra10294a

**Published:** 2020-01-27

**Authors:** Tsun-Ren Chen, Yi-Sheng Lin, Yu-Xiang Wang, Wen-Jen Lee, Kelvin H.-C. Chen, Jhy-Der Chen

**Affiliations:** Department of Applied Chemistry, National Ping Tung University Pingtong City Taiwan trchen@mail.nptu.edu.tw; Department of Applied Physics, National Ping Tung University Pingtong City Taiwan; Department of Chemistry, Chung-Yuan Christian University Chung-Li Taiwan

## Abstract

A catalyst constructed from graphene oxide and iridium chloride exhibited high activity and reliability for the selective transformation of benzylic alcohols into aromatic aldehydes or ketones. Instead of thermal reaction, the transformation was performed under ultrasonication, a green process with low byproduct, high atomic yield and high selectivity. Experimental data obtained from spherical-aberration corrected field emission TEM (ULTRA-HRTEM), X-ray diffraction (XRD), Fourier-transform infrared spectroscopy and Raman spectra confirm the nanostructure of the title complex. Noticeably, the activity and selectivity for the transformation of benzylic alcohols remained unchanged within 25 catalytic cycles. The average turn over frequency is higher than 5000 h^−1^, while the total turnover number (TON) is more than one hundred thousand, making it a high greenness and eco-friendly process for alcohol oxidation.

## Abstract 2

Graphene oxide–iridium nanostructure act as a robust catalyst exhibiting high activity and reliability for the selective transformation of benzylic alcohols into aromatic aldehydes or ketones.

## Graphical abstract

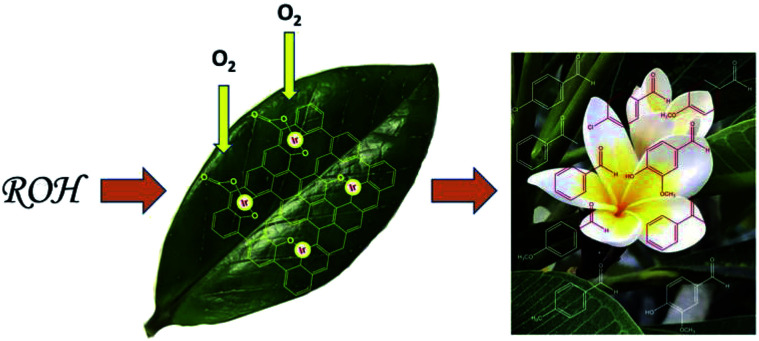

## Introduction

Aromatic aldehydes or ketones are widely used in various fields, such as biological, pharmaceutical, chemical, and materials sciences.^1–5^ A large number of aromatic carbonyl compounds are consumed by certain industries, for example, the global consumption of benzaldehyde is around 7000 tons per year and that of vanillin is estimated to be 12000 tons per year. Therefore, a great effort has been dedicated to developing highly selective and reliable approaches to prepare aromatic aldehydes or ketones in modern synthetic chemistry.^6,7^

There are several ways to prepare aromatic aldehydes or ketones, such as Friedel–Crafts acylation,^8,9^ ozonolysis of alkenes,^10–12^ hydration of alkynes,^13–15^ partial reduction of carboxylic acid derivatives,^16,17^ and the oxidation of alcohols,^18,19^ in which the oxidation of alcohols is the most important method and most widely used, due to the availability of the alcohols. Traditionally, the oxidation of alcohols uses special reagents such as Dess–Martin periodinane,^20,21^ pyridinium chlorochromate (PCC),^22^ permanganate (MnO_4_^1−^),^23^ dichromate (Cr_2_O_7_^2−^) and chromium trioxide (CrO_3_) as oxidants.^24,25^

Most of the above-mentioned methods are well-developed for the oxidation of alcohols to make carbonyl compounds, but all of them use stoichiometric oxidants, and release some toxic by-products, such as heavy metals and chemical wastes. Therefore, tremendous efforts have been devoted to the design of catalytic systems that use O_2_ as the primary oxidant for the catalytic oxidation of benzyl alcohols to prepare benzaldehydes.^26–30^ Although there are some advantages in the new approaches, they could be ameliorated more effectively.

Graphene-based two-dimensional (2D) atomic materials have attracted great attention in the past decade.^31–34^ The single-layered graphene structure as a uniform platform provides a large specific surface area with binding points to interact with substrates and/or active metals, and may form strong π–π interlayer interactions for electrical or energy transfer.^35,36^ Therefore, it has been explored in various fields, such as catalysts,^37–43^ biosensors,^44–48^ supercapacitor,^49–51^ and Li-ion batteries.^52,53^

Iridium named from the Greek goddess of the rainbow possesses some unique characteristics and has been extensively investigated in various fields such as organic light-emitting diode (OLED),^54,55^ solar cells,^56,57^ water splitting to produce hydrogen or oxygen,^58,59^ and carbon dioxide reduction.^60,61^ This element could form compounds with wide range of oxidation states between −3 and +9, resulting in a number of organometallic compounds used in industrial catalysis. For example, the Cativa process for the manufacture of acetic acid,^62,63^ the carbon–hydrogen bond activation for the functionalization of saturated hydrocarbons,^64,65^ and the asymmetric hydrogenation of natural products.^66,67^ Because of the diversity of oxidation state for iridium, the coordination number of iridium atom could flexibly change in iridium complexes; therefore, the complexes would easily undergo reductive elimination to afford a coordinatively unsaturated metal center which could accept substrates and promote some reactions. When iridium atoms landed on the surface of graphene oxide, the functional group of the graphene oxide interacted with iridium atoms to form a graphene–oxide complex with a nano structure. Because of the weak interactions, some donor atoms could departure from the iridium centers to generate open coordination sites on iridium for catalysis. Since the graphene oxides are around the iridium centers, the formation and breakage of the Ir–O and/or Ir–C bonds are reversible and fast. Consequently, the graphene oxide–iridium complex may act as a molecular switch accepting substrates and promoting the catalysis.

Most of chemical reactions were performed at high temperature, and sometimes under high pressure, which would consume a lot of energy and usually bring about side reactions, and result in low selectivity and low transfer yield for desired products. Though catalyst can lower the temperature and improve the selectivity for some reactions, the reaction condition by heating usually lead to catalyst deactivation. In contrast, ultrasonication is usually performed at lower temperatures, which can enhance the efficiency by as much as a million-fold.^68–72^ For a reaction with ultrasonication, it should be noticed that the reactants should be able to efficiently absorb the ultrasonic energy.

Herein, we report a graphene oxide–iridium complex that can be activated by ultrasonic energy, which was applied to the catalytic system for the transformation of benzylic alcohols into aromatic aldehydes or ketones. The preparation and structure of the title complex and the catalytic selectivity and productivity for the target products will be reported.

## Results and discussion

### Catalyst preparation and structural characterization

**Scheme 1:**
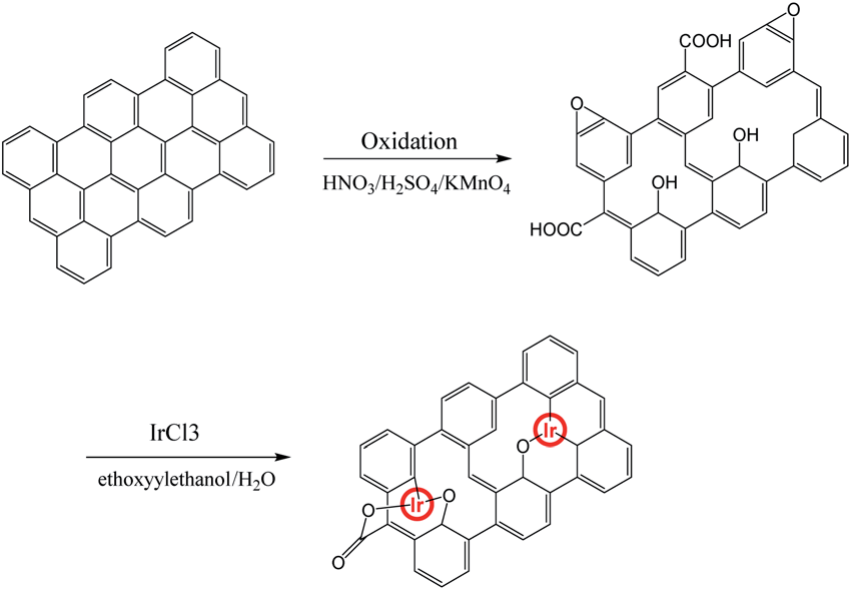
Illustration of the methods used for preparing graphene oxide–iridium complex.

An illustration for generating graphene oxide–iridium complex is shown in Scheme 1. The graphene oxide (GO) was prepared from the direct oxidation of graphite by the method of Hammers. The graphene oxide–iridium complex was synthesized by reacting of GO with iridium chloride in mixed solvent under argon atmosphere.

**Fig. 1:**
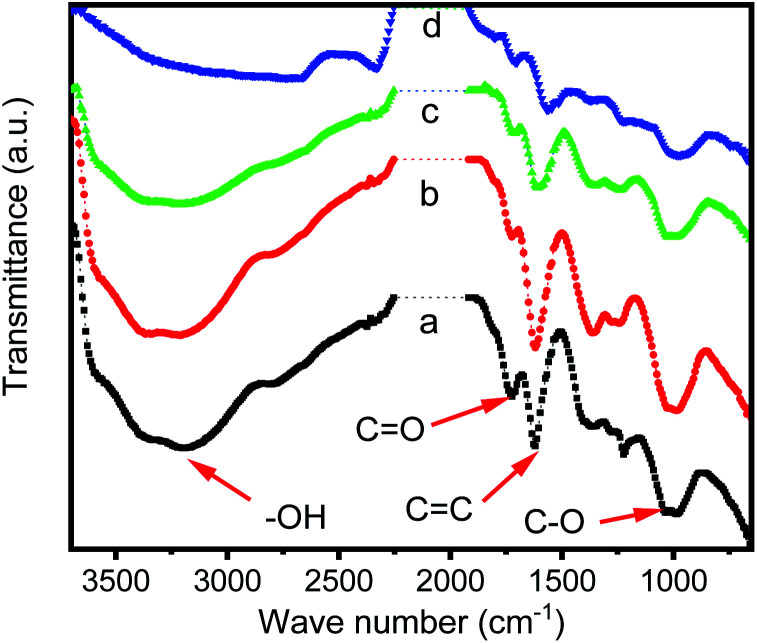
IR spectra of GO and various GO–Ir complexes prepared by the reaction of GO with iridium ion with different reaction time. (a) Original GO, (b) with 2 h, (c) with 6 h, and (d) with 96 h of reaction time, respectively.

Infrared spectra (Fig. 1) showed the progress for the combination of iridium ions with GO. For graphene oxide, some functional groups can be observed on the GO surface, including carboxyl and hydroxyl group (*ν*_O–H_ from 2500 to 3600 cm^−1^), carbonyl group (*ν*_C=O_ at 1723 cm^−1^), C=C stretching (*ν*_C=C_ at 1619 cm^−1^) and C–O stretching (*ν*_C–O_ at 1037 and 978 cm^−1^) (Fig. 1a). When GO was reacting with iridium(iii) chloride, the intensity for *ν*_O–H_ was gradually decreasing and finally almost disappeared after 96 hours of reaction time. The intensities of *ν*_C=O_, *ν*_C=C_ and *ν*_C–O_ were also decreasing but still remained during the reaction. Fig. 1b and c, which reveals that in the reaction progress, GO lose the protons of carboxyl and hydroxyl group, and the conjugate base, carboxylate and alkoxide ions, trapped the iridium ions through the Ir–O interactions. After 96 hours of reacting time (Fig. 1d), most of iridium ions in solution were trapped, and IR spectra do not change further.

**Fig. 2:**
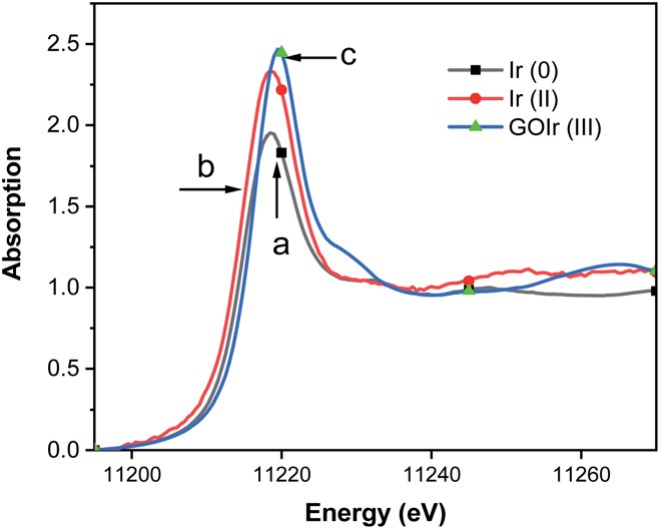
Ir L3-edge XANES of crystalline (a) Ir^0^, (b) [(dfpbo)_2_Ir^II^]_2_, and (c) GO–Ir complex. An Si (111) double crystal monochromator was employed for energy scanning. Fluorescence data were obtained at room temperature using an Ar-filled ionization chamber detector, each sample was scanned 3 times for averaging.

To verify the existence of iridium ions on GO surface, the normalized X-ray absorption near edge structure (XANES) spectra at the Ir K-edge for GO–Ir complex was measured (Fig. 2c) to compare with the spectra of Ir^0^ (Fig. 2a) and [(dfpbo)_2_Ir^II^]_2_ (Fig. 2b). The white line intensity of GO–Ir complex in Fig. 2c shows that the iridium atoms in complex have formal oxidation states higher than Ir^0^ and Ir^II^,^75,76^ which reveal that the iridium ions have been trapped on the GO surface to form GO–Ir complex.

**Fig. 3:**
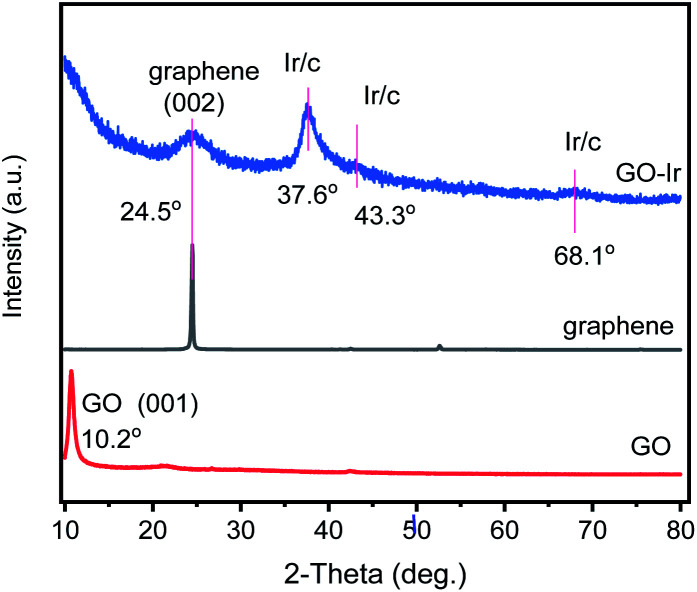
XRD patterns of (bottom) graphene oxide (GO), (middle) graphene, and (top) GO–Ir complex. The XRD pattern of GO shows a characteristic XRD peak of the GO (001) plane. For the XRD pattern of GOIr complex, three new peaks at around 37.6, 43.3, and 68.1° were observed.

The XRD patterns of graphene, synthesized GO, and GO–Ir complex were obtained to realize their fine structures, which were shown in Fig. 3. The characteristic XRD peak of the synthesized GO is at around 2*θ* = 10.2° corresponding to the (001) of GO. For the pattern of GO–Ir complex, the intensity of the XRD peak of GO decreased and a new peak at 2*θ* = 24.5° corresponding to the (002) of graphene was observed, indicating that parts of GO nanostructure were reduced to the graphene structure. It is interesting that there were no typical reduction reagents such as hydride compounds, hydrazine or hydrogen in the reaction condition,^77–79^ but the GO has been reduced to graphene, which could be attributed to the decarboxylation–protonation of carboxyl group, and the dehydration for hydroxyl group on the GO surface. Furthermore, the carboxyl or hydroxyl groups bound to iridium atoms should be stable and the decarboxylation–protonation and dehydration do not occur to them. As shown in Fig. 3, GO–Ir complex exhibits three peaks at around 34.2, 43.6, and 56.8° cannot be attributed to carbon and iridium elements, which were attributed to the characteristic XRD peaks of the GO–Ir complex, indicating the formation of GO–Ir complex.

**Fig. 4:**
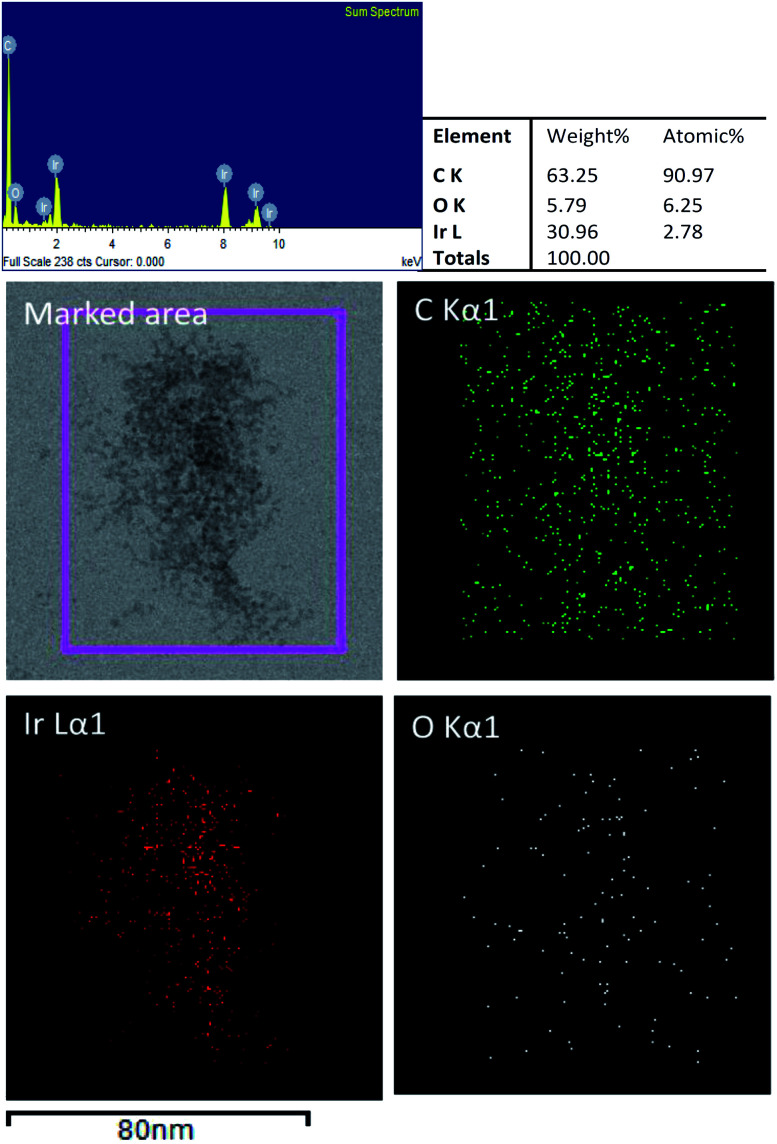
TEM image of GO–Ir complex and elemental mapping images by EDS.

Fig. 4 shows the EDS elemental mappings from a TEM image of GO–Ir, which shows that the image in the marked area was made up of iridium, carbon, and oxygen. The carbon element distribute homogeneously on the marked area, but the oxygen appears mostly at the location where iridium exists, implying that the oxygen atoms are bound to iridium. Quantitative analysis by EDS revealed that the atomic ratio of C, O, and Ir is 90.97 : 6.25 : 2.78, which shows that the atomic ratio of iridium and oxygen is about 1 : 2. By comparing the atomic ratio of carbon with iridium, we can deduce that about 76% of iridium ions were trapped by GO in the reaction condition for preparing graphene oxide–iridium complex.

**Fig. 5:**
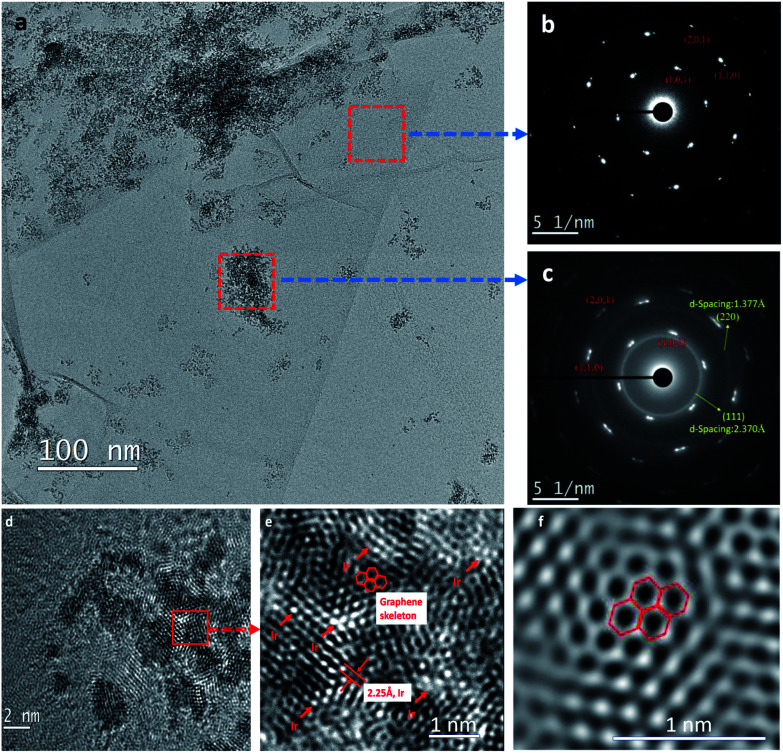
Spherical-aberration corrected field emission TEM images of GO–Ir complex. (a) A low-magnification TEM image, (b) the selected area electron diffraction (SAED) pattern of the surface without iridium atom, (c) the SAED pattern of the surface with iridium atom, (d) high-resolution TEM image, (e) an enlarged fragment taken from (d) (shown by the square symbol), (f) an ultra-high resolution TEM image.

Fig. 5 displays spherical-aberration corrected field emission TEM images of GO–Ir complex. The Fig. 5a demonstrates that GO was successfully exfoliated by ethoxyethanol–water mixed solvent media without using any surfactant, which is a concise process to prepare graphene complex. The selected area electron diffraction (SAED) pattern (Fig. 5b) shows the graphene lattice on the surface of GO–Ir complex without iridium atom, indicating that parts of GO nanostructure were reduced to the graphene structure. The SAED pattern of the surface containing iridium ions (Fig. 5c) shows that there are two *d*-spacing values of 0.237 and 0.137 nm for corresponding to the structure of GO–Ir complex. A representative high-resolution TEM (Fig. 5d) reveals that the grain sizes of iridium range between 0.5 and 5 nm. Fig. 5e is the enlarged fragment taken from Fig. 5d (shown by the square symbol), in which the hexagonal lattice of graphene and iridium atom could be observed, and the corresponding ultra-high resolution TEM image was also shown in Fig. 5f. TEM analysis reveal that the iridium ions were bound to the GO layers to form nanocatalyst. This nanostructure provides a large specific surface area (SSA) and reactive centers for accepting substrates and promoting catalytic reactions.

### Catalysis using GO–Ir complex

**Fig. 6:**
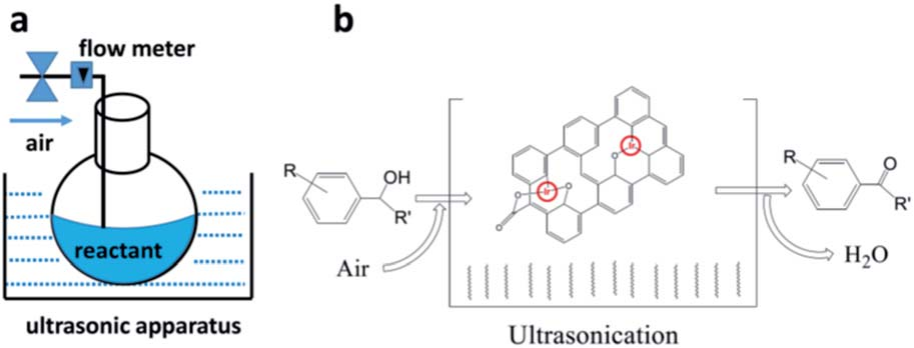
Heterolytic catalysis with ultrasonication for the selective oxidation of benzylic alcohols to produce carbonyl compounds by GO–Ir complex. (a) A heterolytic catalysis by ultrasonication (GO–Ir complex as a heterogeneous catalyst), (b) a scheme showing the reaction.

**Fig. 7:**
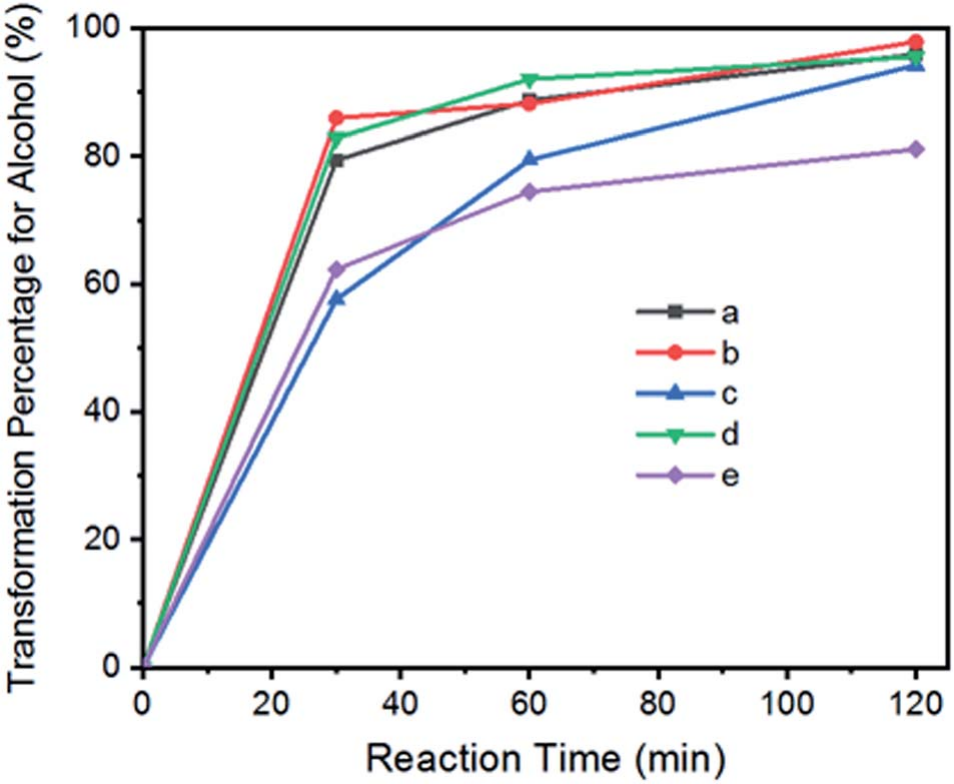
The reaction progress for the catalytic transformation of benzylic alcohols into carbonyl compounds by GO–Ir complex: (a) benzyl alcohol; (b) 4-methylbenzyl alcohol; (c) 4-methoxybenzyl alcohol; (d) 4-chlorobenzyl alcohol; (e) 1-phenyl-2-propanol.

We have previously demonstrated that iridium complexes are highly active catalysts for redox reactions, such as the reduction of carbon dioxide to carbon monoxide,^80^ and the selective oxidation of toluene to benzaldehyde.^81,82^ Here, graphene oxide–iridium complex (GO–Ir complex) was used as a heterogeneous catalyst for a heterolytic catalysis by ultrasonication. The reaction condition is quite simple (Fig. 6a). The benzylic alcohols and GO–Ir complex were mixed in a reaction flask exposed to air and irradiated with ultrasound. During the catalytic reaction, the iridium atoms of GO–Ir complex absorbed the ultrasonic energy (Fig. 6b) to form coordinatively unsaturated metal centers by breaking the Ir–O bonds, which accepted alcohols and promoted the selective transformation of alcohols into carbonyl compounds. The reaction progress was monitored by a high-performance liquid chromatography (HPLC) and GC-MS to characterize the products. Five kinds of aromatic alcohols were used to test the catalytic ability of GO–Ir complex, and all the products are useful in various fields such as agriculture and food industries. The reaction process was shown in Fig. 7, which shows that most of the benzylic alcohols were transformed into the corresponding carbonyl compounds in an hour.

**Fig. 8:**
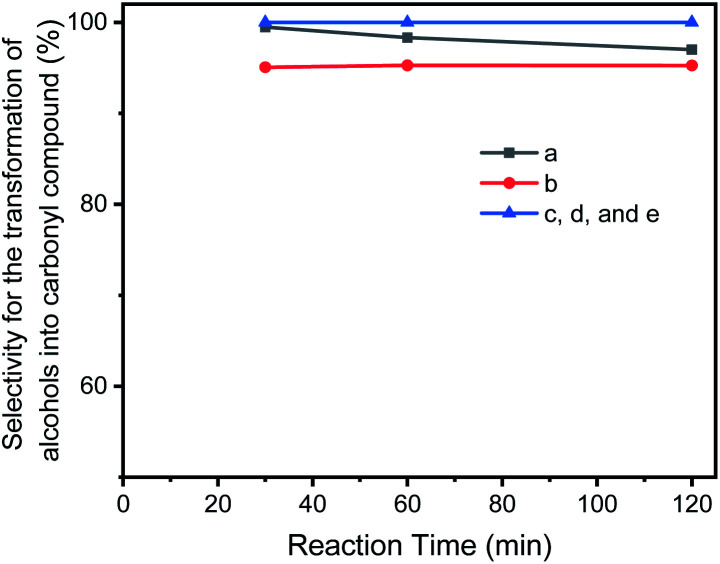
The selectivity for the catalytic transformation of alcohols into carbonyl compounds in reaction progress: (a) benzaldehyde; (b) 4-methylbenzaldehyde; (c) 4-methoxybenzaldehyde; (d) 4-chlorobenzaldehyde; (e) benzylacetone.

Though there was a small by-product observed in the reaction mixtures, the selectivity for the transformation of benzylic alcohols into carbonyl compounds are higher than 96%. In most case, the selectivity achieved 100% (Fig. 8 and Table 1). Table 1 also shows that the atom efficiency for the transformation of alcohols into carbonyl compounds are higher than 98% with an excellent turn over frequency (TOF) (near to 5000 h^−1^). Atom efficiency (atom economy) is an important concept of green chemistry, one of the most widely used metrics for measuring the “greenness” of a process or synthesis, and of importance for human health and the environmental sustainability. The catalysis of GO–Ir complex provides an eco-friendly and market value procedure to transform alcohols into carbonyl compounds. Though for the secondary alcohol, the transformation yield is lower than 90%, the selectivity for it can reach to 100%, and the unreacted reactants can be recovered during the isolation of product. That is, most parts of reactant molecules can be transformed into the target molecules.

**Table 1:** Activity of GO–Ir Complex for the oxidation of benzylic alcohol

Substrate^a^	Product	Conversion^b^ (%)	Selectivity^c^ (%)	Yield^d^ (%)	Atom economy^e^ (%)	TON^f^	TOF^g^ (h^−1^)
Benzyl alcohol	Benzaldehyde (almond oil)	96.24	98.50	95.87	98.15	5982	4956
4-Methybenzyl alcohol	4-Methylbenzaldehyde (cherry scent oil)	98.90	96.24	97.08	98.36	2842	2186
4-Methoxybenzyl alcohol	4-Methoxybenzaldehyde (anise oil)	94.23	100	94.22	98.55	4781	3678
4-Chlorobenzyl alcohol	4-Chlorobenzaldehyde (plant growth regulators)	95.55	100	95.54	98.59	4215	3242
1-Phenyl-2-propanol	Benzylacetone (flowery smell of cocoa)	81	100	81	98.53	5187	3990

a 0.16 M benzylic alcohols in toluene with 0.0005 g GO–Ir complex.

b Transformation percentage for alcohol (%).

c Selectivity for the transformation of alcohol into carbonyl compound.

d Production yield for carbonyl compound.

e Atom economy for the transformation of alcohols into carbonyl compounds without considering the molecular weight of oxygen from the air.

f TON to carbonyl compound after two hour of reaction time.

g TOF (h^−1^) to carbonyl compound.

**Fig. 9:**
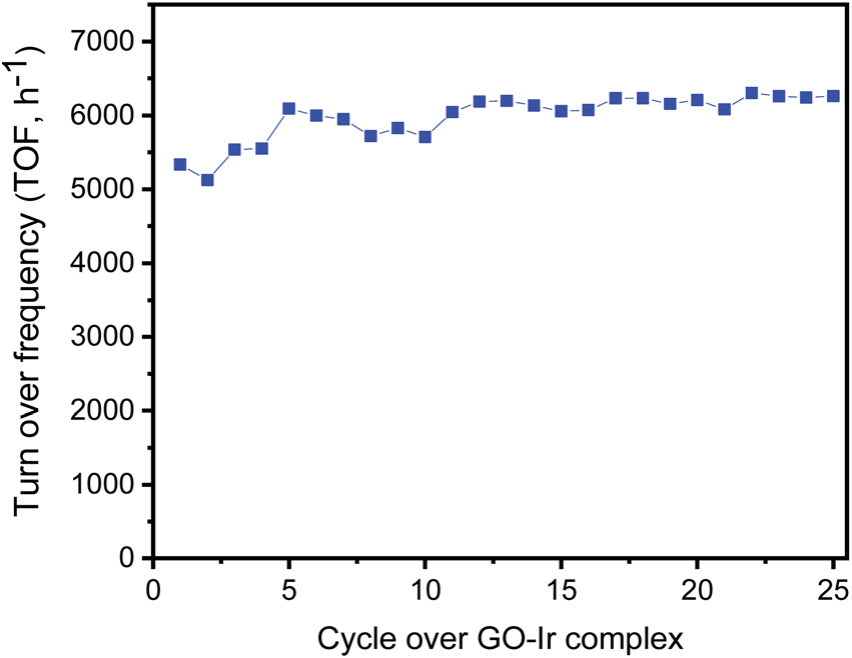
The TOF of the catalytic cycles for the transformation of benzyl alcohol into benzaldehyde, which show that the catalytic capability of GO–Ir complex for the selective transformation of alcohol is quite steady.

The selective transformation of alcohols into carbonyl compounds is an important process for industrial applications, and various catalytic system were developed for this topic, such as Au–Pd nanoparticles on TiO_2_/GO,^83^ Pd nanoparticle encapsulated in a hollow porous carbon sphere,^84^ AuPd core–shell nanoparticles,^85^ and supported gold nanoparticles.^86^ Some of those reports showed good reliability for the catalysts, high transient TOF for catalysis, acceptable TON or selectivity for the transformation of benzylic alcohols, but there are still some challenges to be overcome. A main issue is catalyst deactivations resulting from the destruction of the “non-bonding catalysts”, such as coprecipitated nanoparticles. In contrast, the reactive centers of GO–Ir complex are bound to graphene surface forming a very stable catalytic structure and have a good performance on the catalysis. To evaluate the activity and stability of catalyst in long run production, we studied the catalyst reuse of GO–Ir complex by evaluating the selective oxidation of benzyl alcohol over twenty-five catalytic cycles without a regeneration step (*i.e.*, without washing of catalyst or heat treatment between run). The TOF of the catalytic cycles for the transformation of benzyl alcohol into benzaldehyde was shown in Fig. 9, which showed that the catalytic capability of GO–Ir complex for the selective transformation of alcohol is quite steady, the TOF of the first cycle is 4956 h^−1^, the average TOF within the 25 cycles is about 5020 h^−1^, and the TOF of the latest cycle is 5085 h^−1^. The deactivation is not obvious for GO–Ir complex for the selective transformation of alcohol.

**Fig. 10:**
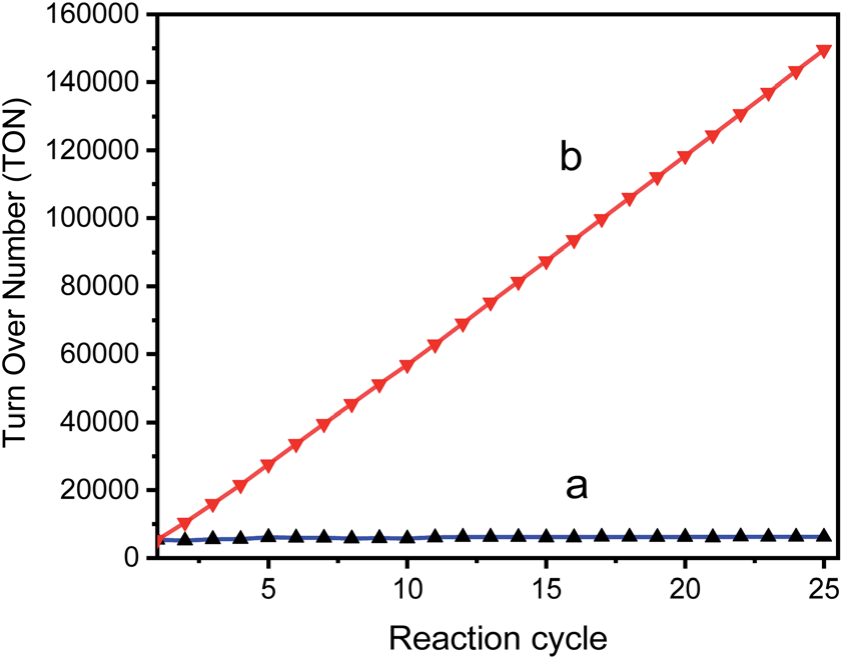
The TON of the catalytic cycles for the transformation of benzyl alcohol into benzaldehyde; (a) TON of each cycle, (b) accumulated TON, for the cycle *n* the accumulated TON was calculated by 
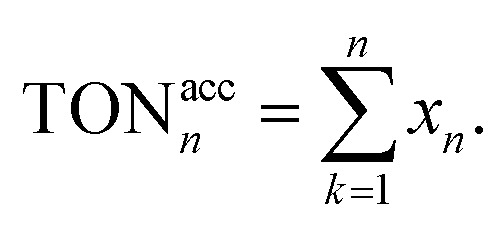

The TON of the catalytic cycles for the transformation of benzyl alcohol into benzaldehyde was also tested by using a 125 ppm solution of GO–Ir complex. The reaction time is 2 h per cycle with ultrasonication (ROCKER SONER 220, 0.5 kW, 53 kHz). The TON of the catalytic cycles for the transformation of benzyl alcohol into benzaldehyde was shown in Fig. 10, revealing that the catalytic capability of GO–Ir complex for the selective transformation of alcohol are fairly stable. The TON of the first cycle is 5334, while the TONs of the other cycles is similar to the first cycle (Fig. 10a), and the average TON within the 25 cycles is about 5982; therefore the TON could be steadily accumulated (Fig. 10b). After 25 cycles, the total TON was more than one hundred thousand (149 500), which imply that the accumulated benzaldehyde productivity attain 233 880 mol_benzaldehyde_ kg_GO−Ir complex_^−1^ within 25 cycles, that is, by using one kilogram of GO–Ir complex, 24 760 kg of benzaldehyde could be produced in 50 hours, which demonstrates a very large productivity. Comparative data for oxidation of benzyl alcohol over reported catalysts and present catalyst is given in the Table 2.

**Table 2:** Comparison of oxidation of benzyl alcohol with reported catalyst

Catalyst	Conversion (%)	Selectivity (%)	TOF (h^−1^)	Run	TON/run	TON (total)	Ref.
AuPd–PVA/TiO_2_	72.5	69.6	34	1	67	67	83
AuPd–PVA/GO	69.5	60.7	32	1	64	64	83
Pd/H_2_Ti_3_O_7_	45.6	70.3	2244	1	4488	4488	86
Pd/TiO2	49.5	76.0	1217	1	2434	2434	86
PMo_11_/Al_2_O	22	91	47	4	4938	19 700	87
PMA/VAMO	32	100	24	1	24	24	88
AuPd/CeO_2_	95	81	30	1	30	30	89
Au–Pd/TiO_2_	72	95.8	589	1			90
Au/TiO2	39	74	6348	1			91
Au/SBA	2	86.1	1937	1	1937	1937	92
GOIr complex	96.2	98.5	5020	25	5982	149 500	This work

**Fig. 11:**
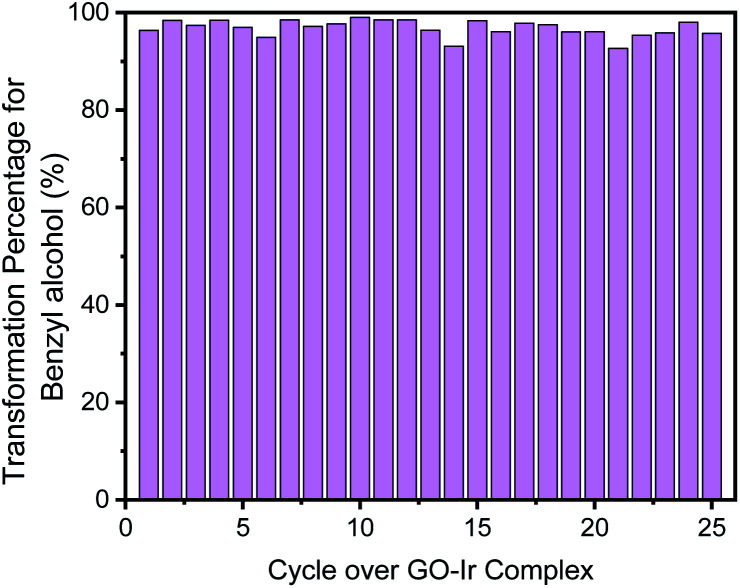
Transformation percentage of each catalytic cycle, which show that most of the benzyl alcohol was transformed.

For each catalytic cycle, most of the benzyl alcohol can be transformed (Fig. 11). The transformation percentage of benzyl alcohol for the first cycle is 96.2%, and the average percentage of transformation within the 25 cycles is 96.8%, which is a process with high efficiency for the transformation of alcohol, that is, most of starting material can be transformed into products, and only a minor amount of reactant should be recovered. It is a time-saving, and energy-saving process.

**Fig. 12:**
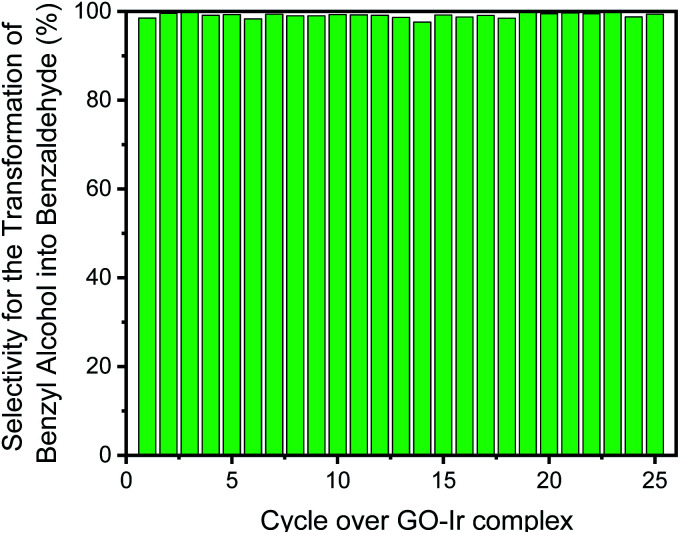
The selectivity of each catalytic cycle, all the catalytic cycle show high selectivity for the transformation of benzyl alcohol into benzaldehyde.

Moreover, the selectivity for the transformation of benzyl alcohol into benzaldehyde is also quite high and steady in each catalytic cycle (Fig. 12). The selectivity for the first cycle is 98.5%, and the average selectivity within the 25 cycles is 99.1%, indicating that most of benzyl alcohol has been transformed into benzaldehyde, and only small amount of by-product (benzoic acid) was observed. The purity of the desired product is very high, and the purification procedure for target compound is quite simple. Because of very low level of waste product forming in the catalytic system, it is a high greenness and eco-friendly strategy for producing carbonyl compounds.

**Fig. 13:**
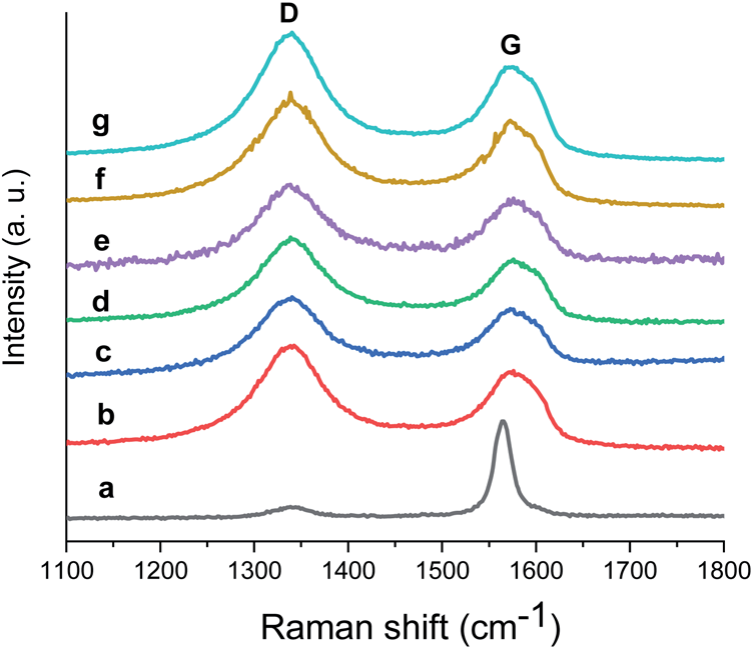
The Raman spectrum of: (a) graphene, (b) fresh GO–Ir complex, (c–g) reused GO–Ir complexes after 1, 5, 10, 20, and 25 catalytic cycles, respectively.

**Fig. 14:**
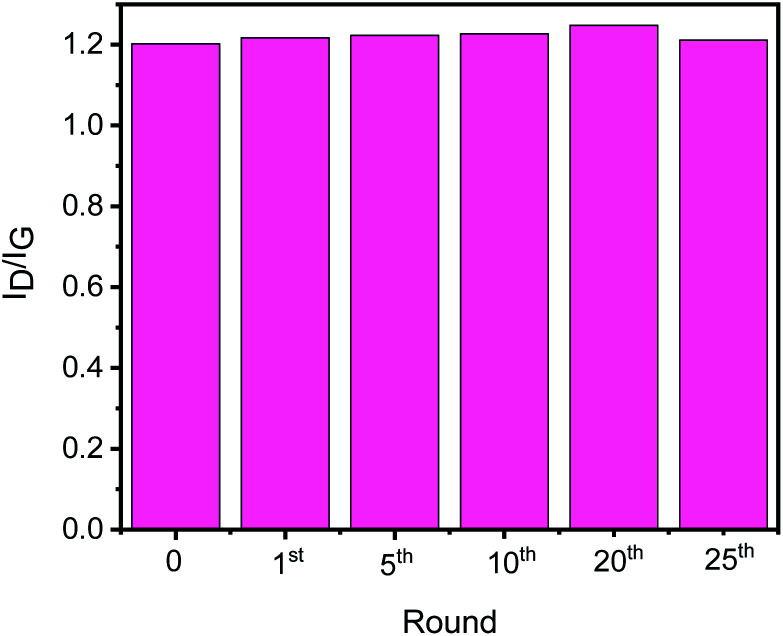
The *I*_D_/*I*_G_ of fresh GO–Ir complex and reused GO–Ir complexes after 1, 5, 10, 20, and 25 catalytic cycles, respectively.

**Fig. 15:**
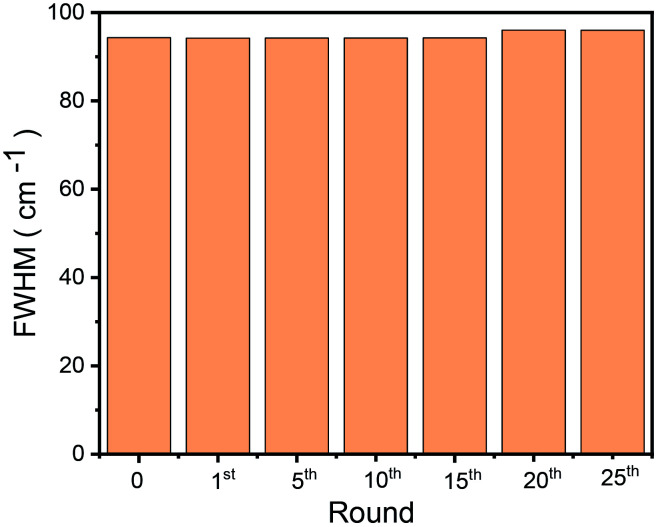
The full width at half maximum (FWHM) of D band for fresh RGOIrNp and reused RGOIrNp after 1, 5, 10, 20, and 25 runs, respectively.

To check the structural stability of GO–Ir complex during catalysis, Raman spectra of original and reused GO–Ir complex were compared by inspecting the characteristics of GO–Ir complex structure. The Raman spectrum of original GO–Ir complex displays a broad D band peak (the vibration of carbon atoms with sp^3^ electronic configuration) at 1340 cm^−1^ and a G band peak (in-plane vibration of sp^2^-bonded carbon atoms) at 1574 cm^−1^ (Fig. 13a). After 25 catalytic cycles, the location of D band and G band peaks of the reused GO–Ir complex keep intact at 1340 and 1574 cm^−1^, respectively (Fig. 13b–g). There was also no significant change in the integrated area under the D band for the reused catalysts. The *I*_D_/*I*_G_ ratio is 1.20 for the original GO–Ir complex, 1.21 for the GO–Ir complex after one run, and 1.21 for that after the 25^th^ run (Fig. 14). Furthermore, there was also no obvious change in the full width at half maximum (FWHM) of D band for GO–Ir complex; the FWHM of original GO–Ir complex is 55.2 cm^−1^. For GO–Ir complex after the 1^st^ and 25^th^ runs, the FWHM are 56.7 and 56.9 cm^−1^, respectively, and the average FWHM within 25 catalytic cycles is 56 cm^−1^ (Fig. 15), which implies that the bonding of GO–Ir complex structure is quite stable, and GO–Ir complex is a robust catalyst.

## Experimental

### Materials

Iridium chloride (IrCl_3_, anhydrous) was obtained from the Seedchem Co. Crystalline graphite was purchased from SHOWA Co. All other chemicals including ethoxyethanol were purchased from Acros and used as received. Aqueous solutions were prepared with double-distilled water from a Millipore system (>18 MΩ cm).

### Preparation of graphene oxide

Graphene oxide (GO) was synthesized from graphite powder following a modified Hummers' method.^73,74^ The synthesized GO was dispersed in DI water (0.5 mg ml^−1^) under ultrasonically treatment for 40 min, and then the solution was heated in an oil bath (90 °C) for 60 min. The obtained GO was filtered through a nylon microporous membrane (0.22 μm) and dispersed in DI water.

### Preparation of graphene oxide–iridium complex

A solution prepared by dissolving 0.3 g of iridium chloride in 50 ml of mixed solvent (ethoxylethanol : water = 3 : 1, v/v) was added to the 100 ml of GO solution (3 mg ml^−1^). The mixture solution was stirred at room temperature for 0.5 h and ultrasonicated for 0.5 h, and then the mixture was refluxed for 96 hours under argon. The obtained graphene oxide–iridium complex dispersion was purified by filtration and washing with DI water and ethanol and then redispersed in ethanol.

### Characterizations

NMR spectra were measured on a Bruker AVIIIHD-600 MHz or a Mercury 300 MHz NMR spectrometer. UV-vis spectra were obtained using a Hitachi U-3900 Spectrophotometer. The infrared spectra were recorded on Agilent Technologies Model Cary 630 FTIR instruments. Mass spectra were taken with a Finnigan/Thermo Quest MAT 95XL instrument with electron impact ionization for organic compounds or fast atom bombardment for metal complexes. Transmission electron microscopy (TEM) images were carried out on a JEOL JEM-ARM200FTH microscopy operated at 80 kV with cold field emission gun (CFEG), spherical-aberration corrector, and high angle annual dark field detector. For the TEM resolution: point image resolution is 0.19 nm, lattice image resolution is 0.10 nm, information limit is 0.10 nm, bright-field lattice image resolution is 0.136 nm, and dark-field lattice image resolution is 0.08 nm, respectively. Energy-dispersive X-ray spectroscopy (EDS) was also performed on the TEM. Raman spectra (RAMaker Raman spectrometer) with an excitation laser of 532 nm were also used to characterize the samples. X-ray Absorption Near-Edge spectroscopy (XANES) was measured on equipment from the National Synchrotron Radiation Research Center (NSRRC, Taiwan). An Si (111) double crystal monochromator was employed for energy scanning. Fluorescence data were obtained at room temperature using an Ar-filled ionization chamber detector, where each sample was scanned 3 times for averaging.

### Catalytic activity of catalysts

The reaction temperature was well controlled in a water bath under a constant temperature (±1 °C). For the catalytic reaction, 0.05–1 g of benzylic alcohol, 0.0005–0.01 g of GO–Ir complex, and 3 ml of toluene were mixed in a reaction flask irradiated with ultrasound, and the reaction progress was monitored by a high-performance liquid chromatography (HPLC) and GC-MS to identify the product composition.

### Catalyst reuse studies

To check the catalytic activity of reused GO–Ir complex, 1 g of aromatic alcohol, 0.0005 of GO–Ir complex, and 3 ml of toluene were mixed in a reaction flask irradiated with ultrasound. After 2 h of reaction time, the reaction mixture was centrifuged to separate out the catalyst, the residual clean supernatant was analyzed by HPLC and GC-MS to identify the product composition. And then, 1 g of aromatic alcohol and 3 ml of toluene was added to a flask containing the recovered GO–Ir complex for the next catalytic cycle. The GO–Ir complex was recovered and used again for 25 times without any evident loss of catalytic activity.

## Conclusions

A robust catalyst constructed from graphene oxide and iridium chloride absorb ultrasonic energy to create active site on the graphene oxide surface, which could accept substrate and selectively transform benzylic alcohols into carbonyl compounds. The transfer yield and selectivity can reach 99 and 100%, respectively. The productivity of carbonyl compounds is 233 880 mol_benzaldehyde_ kg_GO−Ir complex_^−1^ within 25 cycles, during which the structure of the complex was stable, and the capability and selectivity retained consistent. As catalysis was performed at low reaction temperature with ultrasonication, the over-oxidation was suppressed obviously with very little waste produced, demonstrating a high greenness and eco-friendly process for alcohol oxidation.

## Conflicts of interest

There are no conflicts to declare.

## Supplementary Material
